# High posterior cerebral artery flow predicts ischemia recurrence in patients with internal carotid artery occlusion

**DOI:** 10.3389/fneur.2023.1193640

**Published:** 2023-07-20

**Authors:** Simon Jud, Robert Klövekorn, Christiaan Hendrik Bas van Niftrik, Lisa Herzog, Martina Sebök, Juliane Schweizer, Andreas R. Luft, Jorn Fierstra, Susanne Wegener

**Affiliations:** ^1^Department of Neurology, University Hospital Zurich and University of Zurich, Zurich, Switzerland; ^2^Clinical Neuroscience Center, University Hospital Zurich, Zurich, Switzerland; ^3^Department of Neurosurgery, University Hospital Zurich, Zurich, Switzerland; ^4^Institute of Epidemiology, Biostatistics and Prevention, University of Zurich, Zurich, Switzerland; ^5^Stadtspital Waid and Triemli, Zurich, Switzerland; ^6^cereneo Center for Neurology and Rehabilitation, Vitznau, Switzerland

**Keywords:** recurrent stroke, internal carotid artery occlusion, transcranial duplex, posterior cerebral artery, collaterals

## Abstract

Recurrent stroke is a dreaded complication of symptomatic internal carotid artery occlusion (ICAO). Transcranial Duplex (TCD)-derived increased flow velocity in the ipsilateral posterior cerebral artery (PCA)-P2 segment indicates activated leptomeningeal collateral recruitment and hemodynamic impairment. Leptomeningeal collaterals are pial vascular connections between the anterior and posterior vascular territories. These secondary collateral routes are activated when primary collaterals via the Circle of Willis are insufficient. Our goal was to test the TCD parameter PCA-P2 flow for prediction of ipsilateral ischemia recurrence. We retrospectively analyzed clinical and ultrasound parameters in patients with ICAO. Together with clinical variables, we tested systolic PCA-P2 flow velocity as predictor of a recurrent ischemic event using logistic regression models. Of 111 patients, 13 showed a recurrent ischemic event within the same vascular territory. Increased flow in the ipsilateral PCA-P2 on transcranial ultrasound (median and interquartile range [IQR]: 60 cm/s [IQR 26] vs. 86 cm/s [IQR 41], *p* = <0.001), as well as previous transient ischemic attack (TIA) and low NIHSS were associated with ischemia recurrence. Combined into one model, accuracy of these parameters to predict recurrent ischemia was 89.2%. Our data suggest that in patients with symptomatic ICAO, flow increases in the ipsilateral PCA-P2 suggest intensified compensatory efforts when other collaterals are insufficient. Together with the clinical variables, this non-invasive and easily assessable duplex parameter detects ICAO patients at particular risk of recurrent ischemia.

## 1. Introduction

Internal carotid artery occlusion (ICAO) can cause recurrent ipsilateral stroke due to hemodynamic failure of the cerebral collateral supply (1). In the case of ICAO, the primary collateral circulation based on the circle of Willis (CoW) is first activated (Supplementary Figure 1). However, if this primary compensatory route is insufficient, recruitment of secondary collaterals including leptomeningeal vessels occurs, indicating inadequate circulation through the CoW (2). Hence, patients with ICAO often require prolonged observation and intense blood pressure management. Some may even undergo revascularization procedures in cases of recurrent symptoms (3). However, identifying patients at particular risk of recurrent ischemia is challenging. Current methods to detect hemodynamic failure are MRI-based cerebrovascular reactivity (CVR) measurements or (15O)-H2O-positron emission tomography (PET) including a vasodilator stimulus (4–6). Collateral flow in patients with ICAO is accessible through transtemporal insonation using transcranial duplex sonography (TCD). Increased compensatory flow efforts of activated leptomeningeal collaterals result in higher flow velocities in the ipsilateral PCA-P2 segment (7), and have been implicated as indicators of hemodynamic failure (8, 9). Peak systolic flow values >85 cm/s in the ipsilateral PCA-P2 segment indicated hemodynamic failure stage 2 on the affected hemisphere in blood oxygen level dependent (BOLD)-CVR measurements, with an accuracy of 95% (8, 9). Our goal was to assess to what extent TCD-measurements of flow in the ipsilateral PCA-P2, alone or in combination with clinical parameters, predict risk of recurrent ischemic events in symptomatic ICAO patients.

## 2. Materials and methods

### 2.1. Study design and cohort description

This study is a retrospective review of data from patients treated between 2009 and 2019 at the University Hospital Zurich (USZ). The study was approved by the local ethics committees (KEK-ZH: No: 2014-03-04). Patients were included if they were newly diagnosed with an ICAO after suffering an ipsilateral cerebral ischemic event (i.e., ischemic stroke, TIA, retinal infarct, amaurosis fugax), and had received a clinical assessment including an extra- and intracranial duplex investigation before the recurrence ischemia, as well as a follow-up at least 1 month later (see Supplementary methods for inclusion and exclusion criteria). Patients were excluded if they had complete or partial recanalization of the ICAO during hospitalization, were found to have intracranial >50% stenosis or occlusion other than ipsilateral anterior cerebral artery (ACA) and middle cerebral artery (MCA) or a contralateral internal carotid artery (ICA) Stenosis >50% NASCET, or if they did not consent to the scientific use of their data. Recurrent ischemic events were defined as either clinical and/ or radiological evidence of a new focal neurological deficit within the same vascular territory and with no apparent cause other than a new ischemia occurring after the index event. Our goal was to include only ischemia due to hemodynamic impairment in the territory ipsilateral to ICAO. Although some infarct patterns are characteristic for hemodynamic stroke (i.e., internal borderzone infarcts), we refrained from applying strict criteria for infarct morphology, because imaging patterns of embolic stroke have been found in patients with hemodynamic compromise as well (10); and because we also included retinal ischemia and transient ischemic events without infarct demarcation on imaging.

Patients were not specifically screened or selected for fetal variation of the PCA or other collateral variants. Patient characteristics including demographics, clinical data including stroke severity on the NIHSS and modified Ranking Scale (mRS) and medical history prior to stroke were taken from patients charts. Extra- and transcranial duplex sonography was performed on high-end clinical duplex machines, using standardized routine methods. P2 was detected on transtemporal insonation of the axial mesencephalic plane as described previously (11). According to our protocols, no angle correction was applied during flow measurements in the P2 segment.

### 2.2. Statistical analyses

Analyses were performed using descriptive and logistic regression models. Group comparisons were performed using Fisher’s exact test (categorical measurements) and 2-tailed Mann–Whitney *U*-test (continuous measurements). In order to test the variables “previous TIA,” NIHSS on admission and peak systolic flow velocity (PSV) in the ipsilateral PCA-P2-segment (PSV-P2 ipsilateral) for their ability to predict recurrent ischemia, we fit logistic regression models including these variables alone or in combination. A one-fold cross validation approach was used to make use of the entire available data. The parameter estimates from all models were then averaged to obtain a final model for predicting the probability of recurrence for a new patient. The logistic regression model for P different co-variables:


$$\log\left(\frac{P\left(recurrence\right)}{1-P\left(recurrence\right)}\right)=\beta_{0}+\beta_{1}X_{1}+\ldots +\beta_{P}\beta X_{P}$$

with $\beta_{0}$ being the intercept and $\beta_{P}$ the parameters which can be interpreted as log odds ratios for the outcome when the co-variable X is increased by one unit and all other covariables are held constant.

### 2.3. Statement of ethics

This study protocol was reviewed and approved by the KEK-ZH (ethics committee of the canton Zurich, Switzerland), approval number 2014-03-04. Written informed consent was obtained from participants to use routine clinical data; or in cases where patients were unable to give consent: no documented rejection of data use for research was required by the ethical approval.

## 3. Results

**Figure 1:**
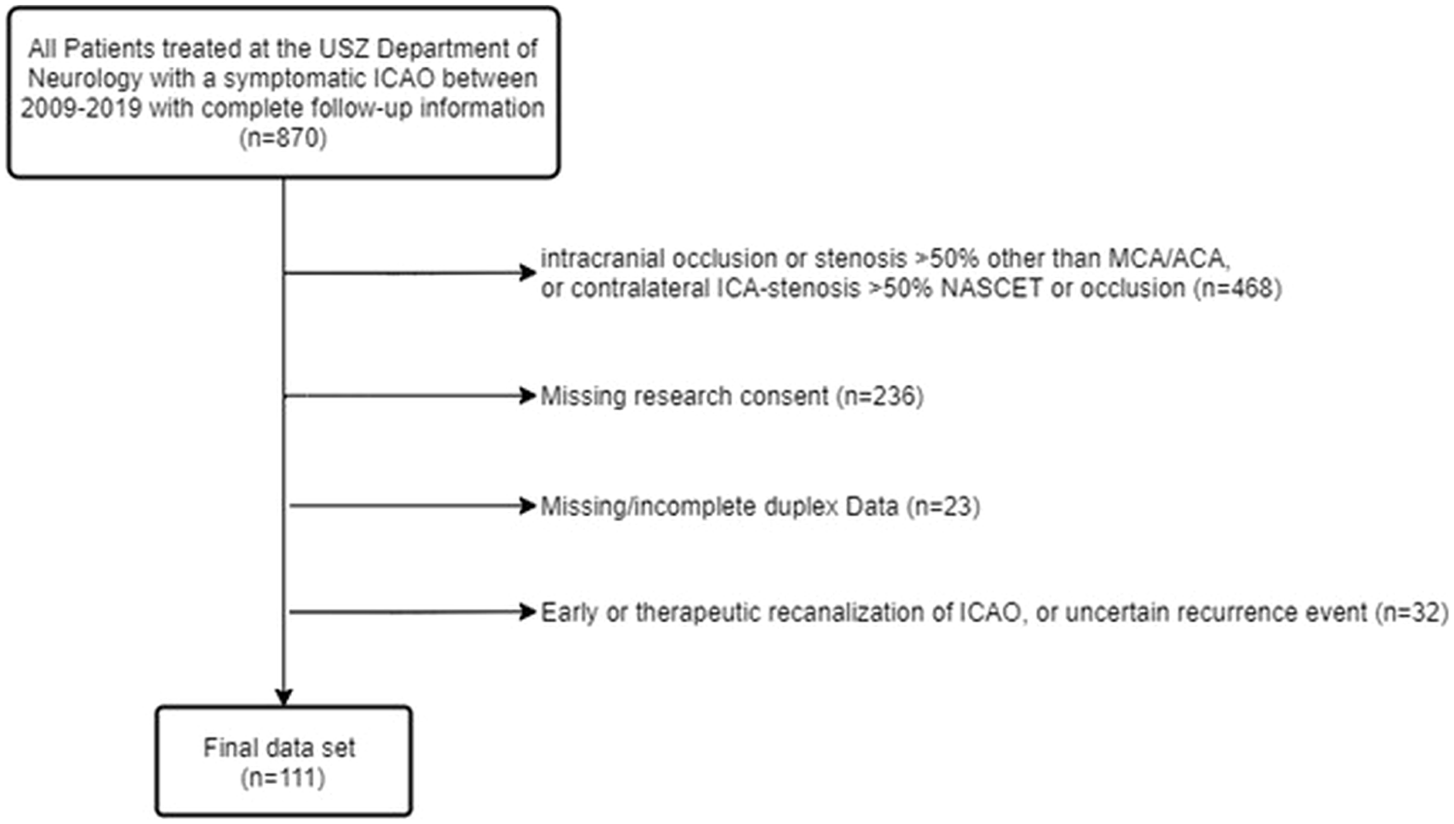
Flow chart of study population. USZ, University Hospital of Zurich; ICAO, internal carotid artery occlusion; MCA, middle cerebral artery; ACA, anterior cerebral artery; NASCET, North American Symptomatic Carotid Endarterectomy Trial.

Out of 870 patients with symptomatic ICAO, we included 111 (see Figure 1). Patients received a TCD at a median time of 1 day after admission [IQR 1], and were observed during a median follow-up of 109 days [IQR 37]. Thirteen (11.7%) patients suffered a recurrent ischemic event within the same vascular territory at a median time of 6 days [IQR 26]. Previous TIA as well as pre-stroke use of anticoagulants and statins were more common in the group with recurrent ischemic event (shown in Table 1). There was no difference in the type of acute stroke treatment between patient with or without recurrent ischemic event. There was a tendency for more atherosclerotic causes (TOAST I) of stroke in the group with recurrence, however, this difference was not significant (*p* = 0.209). In patients with ICAO classified as other determined etiology (TOAST IV), these were carotid artery dissection (*n* = 22), vasculitis (*n* = 1), myeloproliferative disease (*n* = 1), and fibromuscular dysplasia (*n* = 1).

**Table 1:** Patient clinical history, P2-Flow (TCD), stroke etiology and clinical scores.

	All *n* = 111 (%)	No recurrence *n* = 98 (%)	Recurrence *n* = 13 (%)	*p* value
Demographic data				0.209
Age, median years (IQR)	66 (23.5)	66 (23.7)	65 (20.4)	0.962
Female, n (%)	31 (27.9)	26 (26.5)	5 (38.5)	0.511
Medical history and risk factors, n (%)				
Previous stroke	12 (10.8)	9 (9.2)	3 (23.1)	0.148
Previous TIA	14 (12.6)	7 (7.1)	7 (53.8)	**0.001**
Hypertension	77 (69.4)	65 (66.3)	12 (92.3)	0.105
Diabetes	22 (19.8)	18 (18.4)	4 (30.8)	0.458
Dyslipidaemia	66 (59.5)	59 (60.2)	7 (53.8)	0.766
Smoking	37 (33.3)	33 (33.7)	4 (30.8)	1.000
Coronary heart disease	15 (13.5)	12 (12.2)	1 (7.7)	0.380
Peripheral artery disease	11 (9.9)	8 (8.2)	3 (23.1)	0.119
Pre-stroke medication, n (%)				
Antiplatelets	47 (42.3)	40 (40.8)	7 (53.8)	0.552
Anticoagulants	17 (15.3)	11 (11.2)	6 (46.2)	**0.005**
Statins	31 (27.9)	24 (24.5)	7 (53.8)	**0.034**
TCD flow velocity, cm/s, median (IQR)				
Ipsilateral PCA-P2 PSV	62 (26)	60 (26)	86 (41)	**<0.001**
Ipsilateral PCA-P2 EDV	25 (12)	25 (10)	42 (21)	**<0.001**
Stroke etiology, n (%)				0.209
TOAST 1 large artery atherosclerosis	48 (43.2)	39 (39.8)	9 (69.2)	
TOAST 2 cardiac	20 (18)	19 (19.4)	1 (7.7)	
TOAST 3 lacunar	0	0	0	
TOAST 4 other determined	25 (22.5)	24 (24.5)	1 (7.7)	
TOAST 5 undetermined	9 (8.1)	8 (8.2)	1 (7.7)	
TOAST 5 more than one etiology	9 (8.1)	8 (8.2)	1 (7.7)	
Clinical scores, median (IQR)				
NIHSS on admission	4 (11)	5 (12)	1 (3)	**0.005**
Pre-stroke mRS	0 (3)	0 (3)	1 (2)	0.318
mRS 3 months	1 (3)	1 (3)	1 (1)	0.706

Information on patient clinical history, P2-Flow (TCD), stroke etiology and clinical scores (NIHSS on admission, pre- stroke mRS and 3-months-mRS) of all 111 patients and patient groups (no recurrence/ recurrence). Data are expressed as number of patients (n) and percentages or median. *p* values were obtained according to Mann–Whitney *U* test, Pearson’s Chi-squared test and Fisher’s exact test, where appropriate. *p* values < 0.05 are shown in bold. TIA, transient ischaemic attack; PSV, peak systolic velocity; EDV, end diastolic velocity; PCA, posterior cerebral artery.

**Figure 2:**
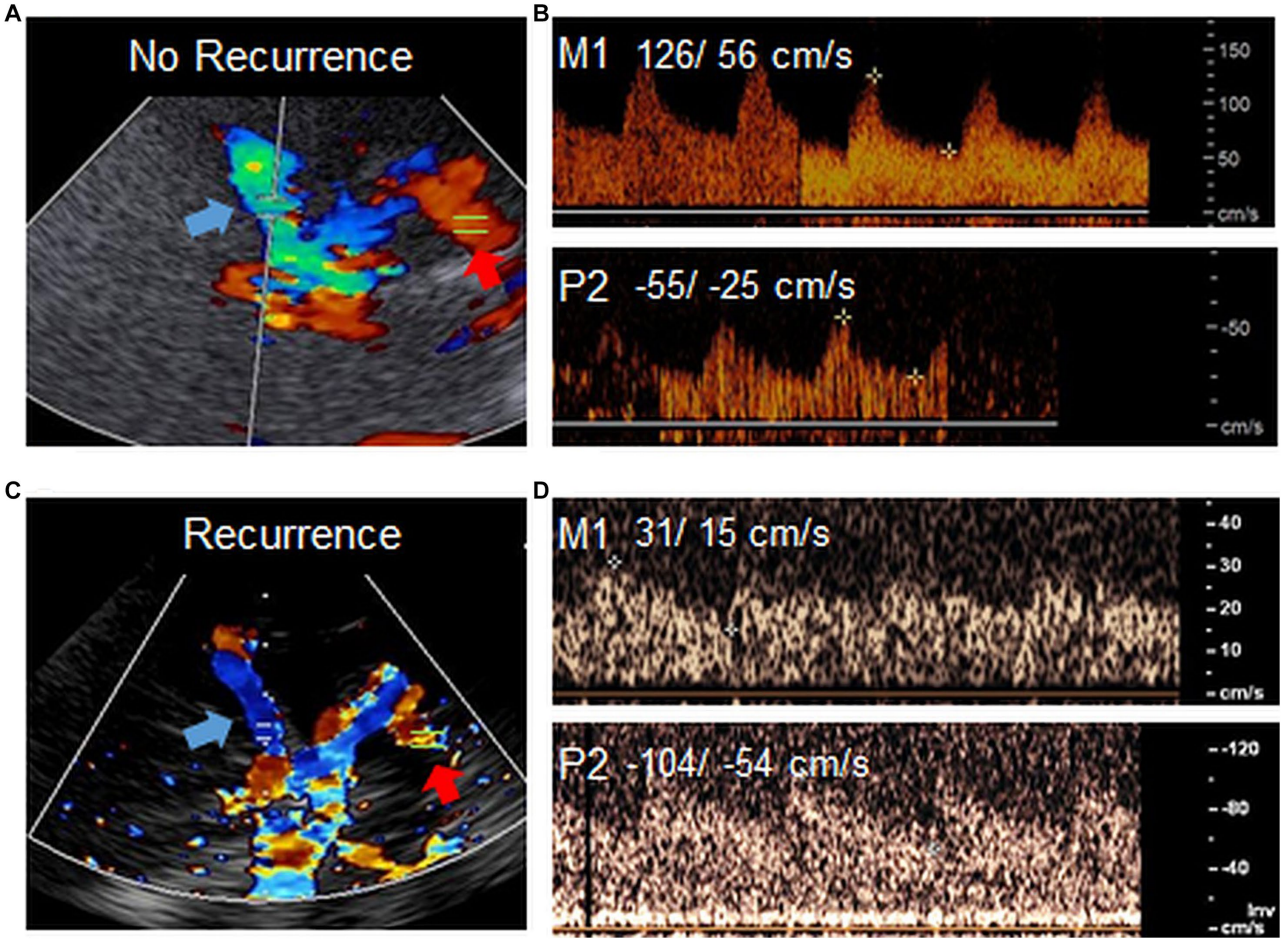
High P2 flow in TCD. Example images of TCD in two patients with ICAO. TCD overview through transtemporal bone window in a patient **(A)** without ischemia recurrence and low flow in the P2 (red arrow) and **(C)** with ischemia recurrence and high flow in the P2 (red arrow). Blue arrows point to the MCA. **(B,D)** Respective doppler flow measurements derived from the ispilateral M1 or P2 in both patients. Flow velocities (PSV/ EDV) are shown in cm/s. The venous signal in the lower panel of **D** points to the basal vein of Rosenthal, which is sometimes detected together with P2 on ultrasound. Note that while in this example, flow in the M1 is particularly low in the patient with ischemia recurrence **(C,D)**, this parameter was not associated with higher risk of recurrent ischemia in our previous study (8). Images were acquired with different ultrasound devices.

Ipsilateral PCA-P2 peak systolic velocity (PSV) values were significantly higher in patients with recurrent ischemia (86 cm/s [IQR 41] vs. 60 cm/s [IQR 26], Figure 2). In addition, the NIHSS on admission was lower in the group with recurrence (1 [IQR 3] vs. 5 [IQR 12]). We found no difference regarding functional disability in both groups on admission or follow up (shown in Table 1).

In order to test the variables “previous TIA,” NIHSS on admission and PSV in the ipsilateral PCA-P2-segment for their ability to predict recurrent ischemia, we fit logistic regression models including these variables alone or in combination (shown in Supplementary Figure 2 and Table 2). PSV in the ipsilateral PCA-P2 segment was indeed a good predictor for recurrent ischemic events (for 86 cm/s: sensitivity of 46.5%, specificity of 91.8%, AUC of 0.79). However, the model including all three co-variables (NIHSS on admission, previous TIA and PSV of the ipsilateral PCA-P2 segment) performed better in predicting ischemia recurrence (accuracy of 89.2% and AUC of 0.91).

**Table 2:** Logistic regression analysis.

Predictor	log(OR)	AUC [CI]	Accuracy
Logistic regression (for each co-variable)			
NIHSS on admission	−0.263	0.68 [0.58, 0.78]	88.29%
Previous TIA	2.72	0.5 [0.24, 0.76]	81.99%
PSV P2 ipsilateral	0.056	0.79 [0.63, 0.94]	88.29%
Logistic regression (for all co-variables)			
Intercept	−7.02	0.91 [0.83, 0.99]	89.19%

Results of the logistic regression analysis for prediction of recurrent ischemic event. The table summarizes the averaged model parameters in terms of the log odds ratios (log(OR)) and the predictive performance of the respective model using the area under the curve (AUC) with corresponding 95% confidence intervals as well as the accuracy for the models including only one variable (either NIHSS on admission, previous TIA, PSV P2 ipsilateral) or all three co-variables together. The model parameters are the averaged model parameters from the 111 iterations from the leave-one-out cross validation.

To exemplify use of this model, in order to predict the probability for ischemia recurrence in a new patient with ICAO, we use:


$$P\left(recurrence\right)=\frac{1}{1+e^{-\left(\beta_{0}+\beta_{1}X_{1}+\ldots +\beta_{P}\beta X_{P}\right)}}.$$

In a patient with a PCA-P2 Flow of 65 cm/s, no previous TIA and an NIHSS admission of 4 would have an estimated risk of ischemia recurrence of 2.5%:


$$\begin{array}{c} P\left(recurrence\right)=\frac{1}{1+e^{-\left(-7.02+0.068*65+2.946*0-0.261*4\right)}}\\ =\frac{1}{1+e^{-\left(-3.644\right)}}=0.025 \end{array}$$

A patient with PCA-P2 Flow of 90 cm/s, previous TIA and an NIHSS admission of 2 would have an estimated risk of ischemia recurrence of 82.1%:


$$\begin{array}{c} P\left(recurrence\right)=\frac{1}{1+e^{-\left(-7.02+0.068*90+2.946*1-0.261*2\right)}}\\ =\frac{1}{1+e^{-\left(1.52\right)}}=0.821. \end{array}$$

## 4. Discussion

Our data show that in patients with symptomatic ICAO, we can forecast the risk of an ipsilateral recurrent ischemic event by history of TIA, a low NIHSS on admission and high flow values in the ipsilateral P2-segment of the PCA with an AUC of 0.91. These results build on existing evidence that increased flow velocity in PCA-P2 after symptomatic ICAO indicates intensified compensatory efforts, associated with risk of hemodynamic failure (8, 9).

In patients with symptomatic ICAO, anterior cerebral artery (ACA) and PCA-P1 segment are considered primary routes of the collateral network (12). P2-supplied leptomeningeal collaterals are considered secondary routes, because they are less efficient (2) (shown in Figure 1). Therefore, increased flow within the P2 segment indicates exhaustion of primary collateral pathways via the CoW as an indicator of hemodynamic compromise.

The correlation of previous TIA with ischemia recurrence was shown previously, highlighting the importance of clinical symptoms in predictive models (8, 13–17). In our sample, patients with a low NIHSS score on admission had a higher risk of recurrence. This appears counterintuitive, since low NIHSS scores indicate less tissue damage (18). One explanation for this finding could be that in severely affected patients, minor recurrent ischemic events due to hemodynamic failure may not cause clinically apparent worsening and thus, may be missed. Since our study included stroke patients with rather minor deficits on admission (median NIHSS of 4) resulting in underrepresentation of a more severe stroke population.

Choosing the best preventive strategy after ischemia has recurred in patients with ICAO is challenging. In our patients, prevention was up to the preference of the treatment team and included addition of a second platelet inhibitor, anticoagulation, or interventional procedures, such as extra-intracranial (EC-IC) -bypass surgery. While our sample size is too small to derive meaningful conclusions about the best treatment concept, our data strongly support that more studies are needed to establish therapeutic recommendations.

Overall, our results support the hypothesis that TCD PCA-P2 measurements can help detecting patients at an increased risk of recurrent ischemic events. TCD is more readily available as a screening tool and can identify high risk ICAO patients that will need a more comprehensive hemodynamic imaging work-up with for instance, BOLD-CVR or (15O-)H2O-PET. However, if used in conjunction with these advanced imaging readouts of CVR, TCD and clinical variables are likely to further increase prediction accuracy, which should be validated in prospective studies. Several variants of the CoW exist in healthy subjects as well as in patients with stroke. While we did not detect an association of CoW variants with outcome in patients with stroke due to MCA-M1 occlusion previously (19), such variants may well affect flow in the PCA-P2 segment. However, even without further consideration of CoW variants in our study cohort, P2 flow was highly associated with recurrent hemodynamic stroke in our study. This facilitates use of this easily derived non-invasive TCD parameter for the detection of hemodynamic impairment in stroke patients. One limitation of our analysis is the small patient cohort. Furthermore, the strict exclusion of patients with other moderate or high-grade intra- or extracranial vessel pathology limits generalizability of our findings. Validation in a larger, multicenter cohort would improve generalizability of our findings. Standardization of TCD measurements is required to reduce examiner-dependent variability. However, if angle correction is omitted, interrater reliability of TCD flow assessment in the PCA-P2 is high.

To conclude, in patients with symptomatic ICAO, increased ipsilateral TCD PCA-P2 flow velocity together with history of TIA and low admission NIHSS indicate an increased risk of recurrent ischemia with high accuracy. TCD can aid in non-invasive assessment of hemodynamic impairment, and should be included into clinical algorithms to improve detection of patients at risk.

## Data availability statement

Anonymized raw data can be made available from the corresponding author by reasonable request.

## Ethics statement

The studies involving human participants were reviewed and approved by KEK-ZH (Ethics Committee of the Canton Zurich, Switzerland). Written informed consent for participation was not required for this study in accordance with the national legislation and the institutional requirements.

## Author contributions

SW conceived and designed the study. SJ and RK wrote the first draft of the manuscript and were responsible for data collection and data analysis. CN, MS, and JF were involved in concept development and image analysis. LH performed statistical data analysis. AL and JF were involved in interpretation of study data. All authors reviewed and edited the manuscript and approved the final version of the manuscript.

## Funding

This project was supported by the Swiss National Science Foundation (PP00P3_202663), the UZH CRPP Stroke, the Baugarten foundation, and the P&K Pühringer Foundation.

## Conflict of interest

The authors declare that the research was conducted in the absence of any commercial or financial relationships that could be construed as a potential conflict of interest.

## Publisher’s note

All claims expressed in this article are solely those of the authors and do not necessarily represent those of their affiliated organizations, or those of the publisher, the editors and the reviewers. Any product that may be evaluated in this article, or claim that may be made by its manufacturer, is not guaranteed or endorsed by the publisher.
