# Supplementary material for: High posterior cerebral artery flow predicts ischemia recurrence in patients with internal carotid artery occlusion
Source: Front Neurol. 2023 Jul 20;14:1193640. doi: 10.3389/fneur.2023.1193640 (PMC10397388; doi:10.3389/fneur.2023.1193640)
Supplement: Supplementary file 1 [file Data_Sheet_1.docx]

**Supplementary Figure 1**

**
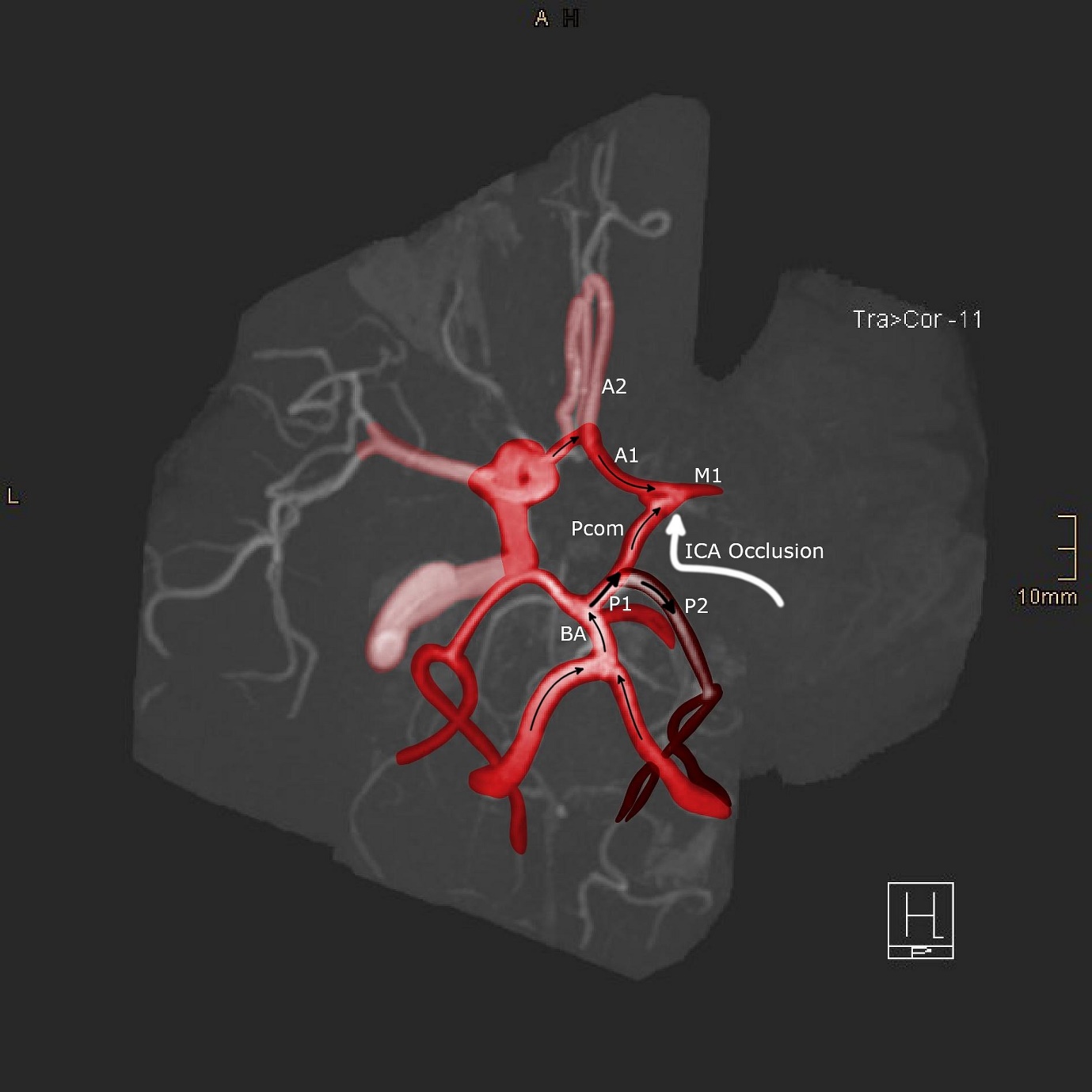
**

**Legend Supplementary Figure 1:** MR Angiography of arterial cerebral vasculature of a patient with occlusion of the left internal carotid artery. The circle of Willis (CoW) and its main branches are colored. Because of the ICAO, cerebral blood flow particularly to the main segment of the middle cerebral artery (M1) is severely restricted. Compensatory flow arrives retrograde via the anterior cerebral artery (A1 & A2) and anterograde via the basilar artery (BA) and the posterior communicating artery (Pcom). TCD measures high flow velocities in the P1 & P2-Segment of the posterior cerebral Artery (P1 & P2).

**Supplementary Figure 2**

**
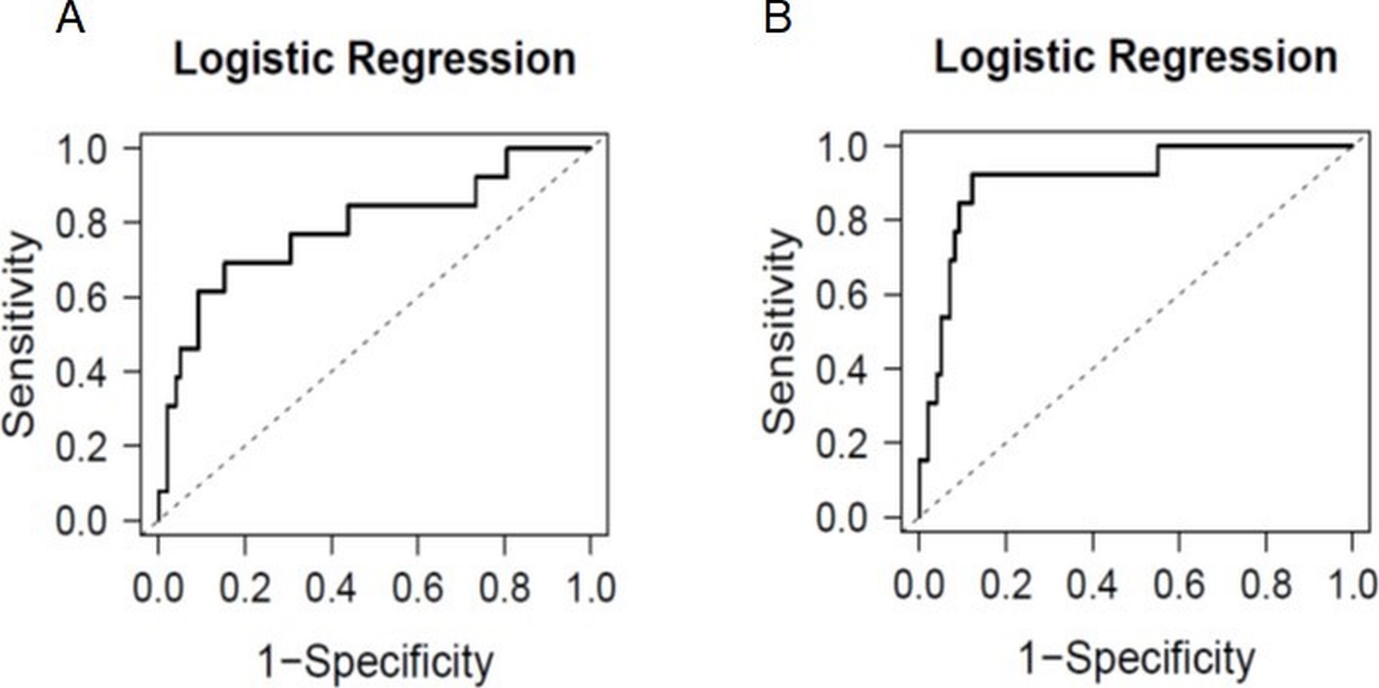
**

**Legend Supplementary Figure 2:** Area under the Curve (AUC) analysis from Receiver Operating Curve (ROC) for A) the model including only PCA-P2 flow, and B) the model including PCA-P2 flow, previous TIA and NIHSS on admission as co-variables.
